# Predicting Outcome of Endovascular Treatment for Acute Ischemic Stroke: Potential Value of Machine Learning Algorithms

**DOI:** 10.3389/fneur.2018.00784

**Published:** 2018-09-25

**Authors:** Hendrikus J. A. van Os, Lucas A. Ramos, Adam Hilbert, Matthijs van Leeuwen, Marianne A. A. van Walderveen, Nyika D. Kruyt, Diederik W. J. Dippel, Ewout W. Steyerberg, Irene C. van der Schaaf, Hester F. Lingsma, Wouter J. Schonewille, Charles B. L. M. Majoie, Silvia D. Olabarriaga, Koos H. Zwinderman, Esmee Venema, Henk A. Marquering, Marieke J. H. Wermer, the MR CLEAN Registry Investigators

**Affiliations:** ^1^Department of Neurology, Leiden University Medical Center, Leiden, Netherlands; ^2^Department of Biomedical Engineering and Physics, University of Amsterdam, Amsterdam, Netherlands; ^3^Department of Clinical Epidemiology and Biostatistics, University of Amsterdam, Amsterdam, Netherlands; ^4^Leiden Institute for Advanced Computer Sciences, Leiden University, Leiden, Netherlands; ^5^Department of Radiology, Leiden University Medical Center, Leiden, Netherlands; ^6^Department of Neurology, Erasmus Medical Center, Rotterdam, Netherlands; ^7^Department of Biomedical Data Sciences, Leiden University Medical Center, Leiden, Netherlands; ^8^Department of Public Health, Erasmus Medical Center, Rotterdam, Netherlands; ^9^Department of Radiology, University Medical Center Utrecht, Utrecht, Netherlands; ^10^Department of Neurology, Antonius Ziekenhuis, Nieuwegein, Netherlands; ^11^Department of Radiology and Nuclear Medicine, University of Amsterdam, Amsterdam, Netherlands

**Keywords:** ischemic stroke, prediction, machine learning, endovascular treatment, functional outcome, reperfusion

## Abstract

**Background:** Endovascular treatment (EVT) is effective for stroke patients with a large vessel occlusion (LVO) of the anterior circulation. To further improve personalized stroke care, it is essential to accurately predict outcome after EVT. Machine learning might outperform classical prediction methods as it is capable of addressing complex interactions and non-linear relations between variables.

**Methods:** We included patients from the Multicenter Randomized Clinical Trial of Endovascular Treatment for Acute Ischemic Stroke in the Netherlands (MR CLEAN) Registry, an observational cohort of LVO patients treated with EVT. We applied the following machine learning algorithms: Random Forests, Support Vector Machine, Neural Network, and Super Learner and compared their predictive value with classic logistic regression models using various variable selection methodologies. Outcome variables were good reperfusion (post-mTICI ≥ 2b) and functional independence (modified Rankin Scale ≤2) at 3 months using (1) only baseline variables and (2) baseline and treatment variables. Area under the ROC-curves (AUC) and difference of mean AUC between the models were assessed.

**Results:** We included 1,383 EVT patients, with good reperfusion in 531 (38%) and functional independence in 525 (38%) patients. Machine learning and logistic regression models all performed poorly in predicting good reperfusion (range mean AUC: 0.53–0.57), and moderately in predicting 3-months functional independence (range mean AUC: 0.77–0.79) using only baseline variables. All models performed well in predicting 3-months functional independence using both baseline and treatment variables (range mean AUC: 0.88–0.91) with a negligible difference of mean AUC (0.01; 95%CI: 0.00–0.01) between best performing machine learning algorithm (Random Forests) and best performing logistic regression model (based on prior knowledge).

**Conclusion:** In patients with LVO machine learning algorithms did not outperform logistic regression models in predicting reperfusion and 3-months functional independence after endovascular treatment. For all models at time of admission radiological outcome was more difficult to predict than clinical outcome.

## Introduction

Endovascular treatment (EVT) is effective for ischemic stroke patients with a large vessel occlusion (LVO) of the anterior circulation. EVT results in a number needed to treat of 2.6 to reduce disability by at least one level on the modified Rankin Scale (mRS) (1). A recent meta-analysis showed a positive treatment effect of EVT across patient subgroups including different age groups, varying stroke severity, sex, and stroke localization (1). However, many clinical and imaging predictors or their combinations were not considered in the subgroup analysis. Moreover, the RCTs that provided the data differed in their patient selection criteria. To further improve personalized stroke care, it is essential to accurately predict outcome and eventually differentiate between patients who will and will not benefit from EVT.

Machine learning belongs to the domain of artificial intelligence and provides a promising tool in pursuing personalized outcome prediction, which is increasingly used in medicine (2–7). The machine learning methodology allows discovering empirical patterns in data through automated algorithms. In some clinical settings machine learning algorithms outperform classical regression models, such as logistic regression, possibly through more efficient processing of non-linear relationships and complex interactions between variables (6, 8), although poorer performance has also been observed (9).

In this study, we used multiple machine learning algorithms and logistic regression with multiple variable selection methods to predict radiological and clinical outcome after EVT in a cohort of well-characterized stroke patients. We hypothesized that machine learning algorithms outperform classic multivariable logistic regression models in terms of discrimination between good and poor radiological and clinical outcome.

## Methods

### Patients

We included patients registered between March 2014 and June 2016 in the Multicenter Randomized Clinical Trial of Endovascular Treatment for Acute Ischemic Stroke in the Netherlands (MR CLEAN) Registry. The MR CLEAN Registry is an ongoing, national, prospective, open, multicenter, observational monitoring study covering all 18 stroke intervention centers that perform EVT in the Netherlands, of which 16 participated in the MR CLEAN trial (10). The registry is a continuation of the MR CLEAN trial collaboration and includes all patients undergoing EVT (defined as entry into the angiography suite and receiving arterial puncture) for acute ischemic stroke in the anterior and posterior circulation. In the current analysis we included those patients who adhered to the following criteria: age 18 years and older, treatment in a center that participated in the MR CLEAN trial, and LVO in the anterior circulation (internal carotid artery (ICA), internal carotid artery terminus (ICA-T), middle (M1/M2) cerebral artery, or anterior (A1/A2) cerebral artery), shown by CT angiography (CTA) or digital subtraction angiography (DSA) (11).

### Clinical baseline characteristics

We assessed the following clinical characteristics at admission: National Institutes of Health Stroke Scale (NIHSS), Glasgow Coma Scale, medical history (TIA, ischemic stroke, intracranial hemorrhage, subarachnoid hemorrhage, myocardial infarction, peripheral artery disease, diabetes mellitus, hypertension, hypercholesterolemia), smoking, laboratory tests (blood glucose, INR, creatinine, thrombocyte count, CRP), blood pressure, medication (thrombocyte aggregation inhibitors, oral anticoagulant drugs, anti-hypertensive drugs, statins), modified Rankin Score (mRS) before stroke onset, administration of intravenous tPA (yes/no), stroke onset to groin time, transfer from another hospital, and whether the patient was admitted during weekend or off hours.

### Radiological baseline parameters

All imaging in the MR CLEAN Registry was assessed by an imaging core laboratory (11). On non-contrast CT, the size of initial lesion in the anterior circulation was assessed by the Alberta Stroke Program Early CT Score (ASPECTS). ASPECTS is a 10 point quantitative topographic score representing early ischemic change in the middle cerebral artery territory, with a scan without ischemic changes receiving an ASPECTS of 10 points (12). In addition, presence of leukoaraiosis and old infarctions, hyperdense vessel sign, and hemorrhagic transformation of the ischemic lesion were assessed on non-contrast CT.

On CTA, the core lab determined clot burden score, clot location, collaterals, and presence of intracranial atherosclerosis. The clot burden score evaluates the extent of thrombus in the anterior circulation by location scored on a 0–10 scale. A score of 10 is normal, implying clot absence; a score of 0 implies complete multi-segment vessel occlusion (12). Presence of intracranial carotid artery stenosis, atherosclerotic occlusion, floating thrombus, pseudo-occlusion, and carotid dissection were scored on CTA of the carotid arteries. Collaterals were assessed using a 4 point scale, with 0 for absent collaterals (0% filling of the vascular territory downstream of the occlusion), 1 for poor collaterals (>0% and ≤50% filling of the vascular territory downstream of the occlusion), 2 for moderate collaterals (>50% and <100% filling of the vascular territory downstream of the occlusion), and 3 for excellent collaterals (100% filling of the vascular territory downstream of the occlusion) (13).

### Treatment specific variables

Variables collected during EVT were type of sedation during the procedure (general or conscious), use of a balloon guiding catheter, carotid stent placement, performed procedure (DSA only or thrombectomy), and type of EVT-device (stent retriever, aspiration device, or a combination of both). In addition, data were collected on adverse events during the procedure (perforation, dissection, distal thrombosis on DSA).

Interventional DSA parameters in our dataset were occluded vessel segment (ICA: origin, cervical, petrous, cavernous, supraclinoid, M1-M4, A1, A2), arterial occlusive lesion (AOL) recanalization score before and after EVT (14), evidence of vascular injury (i.e., perforation, or dissection, vasospasm, new clot in different vascular territory or distal thrombus confirmed with imaging), and modified Thrombolysis in Cerebral Infarction (mTICI)-score before and after EVT. The mTICI-score grades the following categories of cerebral reperfusion: no reperfusion of the distal vascular territory (0), minimal flow past the occlusion but no reperfusion (1), minor partial reperfusion (2a), major partial reperfusion (2b), and complete reperfusion (3) (15). Further variables analyzed were time from stroke onset to recanalization, post-EVT stay on intensive care, high care or stroke care, NIHSS after EVT (<48 h), delta NIHSS (pre-treatment NIHSS subtracted from NIHSS <48 h after EVT) and hemicraniectomy or symptomatic intracranial hemorrhage <48 h after EVT.

### Outcome

The primary radiological outcome was good reperfusion defined as modified TICI-score directly post-procedure (post-mTICI) ≥ 2b (15). The primary clinical outcome was functional independence at 3 months after stroke (mRS ≤ 2). We excluded patients in whom any of the main outcomes (3-months mRS and post-mTICI) were missing.

To investigate the full potential of Machine learning compared with conventional methods in different settings after stroke we defined two prediction settings:

First, we assessed the probability of good reperfusion and good 3-months functional independence in our cohort of patients that underwent EVT based only on variables that were available on admission before entry into the angiography suite. With this baseline prediction setting we are able to investigate the added value of machine learning for models that could potentially support future clinical decision making regarding the performance of EVT yes or no.

Second, we tested the models for predicting 3-months functional independence in patients after EVT was performed. For this analysis we used all variables collected up to 48 h after the end of the endovascular procedure (baseline and treatment variables).

### Machine learning algorithms

The machine learning algorithms used in our study were Random Forests, Artificial Neural Network and Support Vector Machine, because they are among the algorithms that are currently most widely and successfully used for clinical data (2–7). Each one of them represents a different algorithm “family,” each with radically different internal algorithm structures (16). Since it was not known beforehand which kind of algorithm would perform best, we chose algorithms with different internal structures to increase the probability of good discriminative performance. We also used Super Learner, which is an ensemble method that finds the optimal weighted combination of predictions of the Random Forests, Artificial Neural Network and Support Vector Machine algorithms used in this study. Ensemble methods, such as Super Learner have been shown to increase predictive performance by increasing model flexibility (17). For the implementation of all machine learning algorithms we used off-the-shelf methods in the Python module Scikit-Learn (18).

#### Super learner

Super Learner is a stacking algorithm using cross-validated predictions of other models (i.e., a machine learning algorithm and logistic regression) and assigning weights to these predictions to optimize the final prediction. Super Learner's predictive performance has been found to surpass individual machine learning models in various clinical studies (17, 19, 20).

#### Random forests

Random Forests consists of a collection of decision tree classifiers that are fit on random subsamples of patients and variables in the dataset. The variation of the subsampled variables creates a robust classifier. In the decision trees, each node represents a variable and splits the input data into branches based on an objective function that determines the optimal threshold for separating the outcome classes. The predictions from each tree are used as “votes,” and the outcome with the most votes is considered the predicted outcome for that specific patient (6, 21). From the Random Forests algorithm variable importances can be derived, which are the sum of weights of nodes of the trees containing a certain variable, averaged over all trees in the forest (22).

#### Support vector machine

Support Vector Machine (SVM) is a kernel-based supervised machine learning classifier which can also be used to output probabilities. The SVM works by first mapping the input data into a high dimensional variable space. For binary classification, a hyperplane is subsequently determined to separate two classes such that the distance between the hyperplane and the closest data points is maximized (23).

#### Artificial neural network

In this study we use the multilayer perceptron, a popular class of artificial neural network architecture composed of one or more interconnected layers of neurons that process data from the input layer into predictions for the output layer. The algorithm computes a weight for each neuron based on input activation. These weights are updated by backpropagation and stochastic gradient descent (24, 25).

### Logistic regression

For logistic regression, generally a set of variables has to be selected to be included in the model. Since model performance can rely heavily on selecting the right variables, we tested five different variable selection methods prior to logistic regression. We first selected variables based on prior knowledge, a still widely used method that could be considered “classical” (26). We selected 13 variables available at baseline that were included in a previous study for a similar purpose (27) (Supplementary Table Ia). In addition, from baseline and treatment variables we selected 15 variables based on expert opinions of vascular neurologists and radiologists (Supplementary Table Ib).

We further considered four automated variable selection methods: (i) backward elimination, which is also considered to be a more classical approach (26), and three state-of-the-art variable selection methods: (ii) least absolute shrinkage and selection operator (LASSO) (28), (iii) Elastic Net, which is a modification of the LASSO found to outperform the former while still having the advantage of a similar sparsity of representation (29), and (iv) selection based on Random Forests variable importance.

### Analysis pipeline

We imputed missing values using multiple imputations by chained equations (MICE) (30). Variables with 25% missing values or more were discarded from further analysis. All remaining variables used in this study are listed in Supplementary Tables II, III. In total, 53 baseline variables and 30 treatment variables were used as input for machine learning algorithms and automated variable selection methods for logistic regression.

The ordinal clinical (NIHSS) and radiological (clot burden and ASPECTS) scores were presented as continuous scores in all models to increase model efficiency, and we assumed linear trends underlying the ordinal scores.

**Figure 1:**
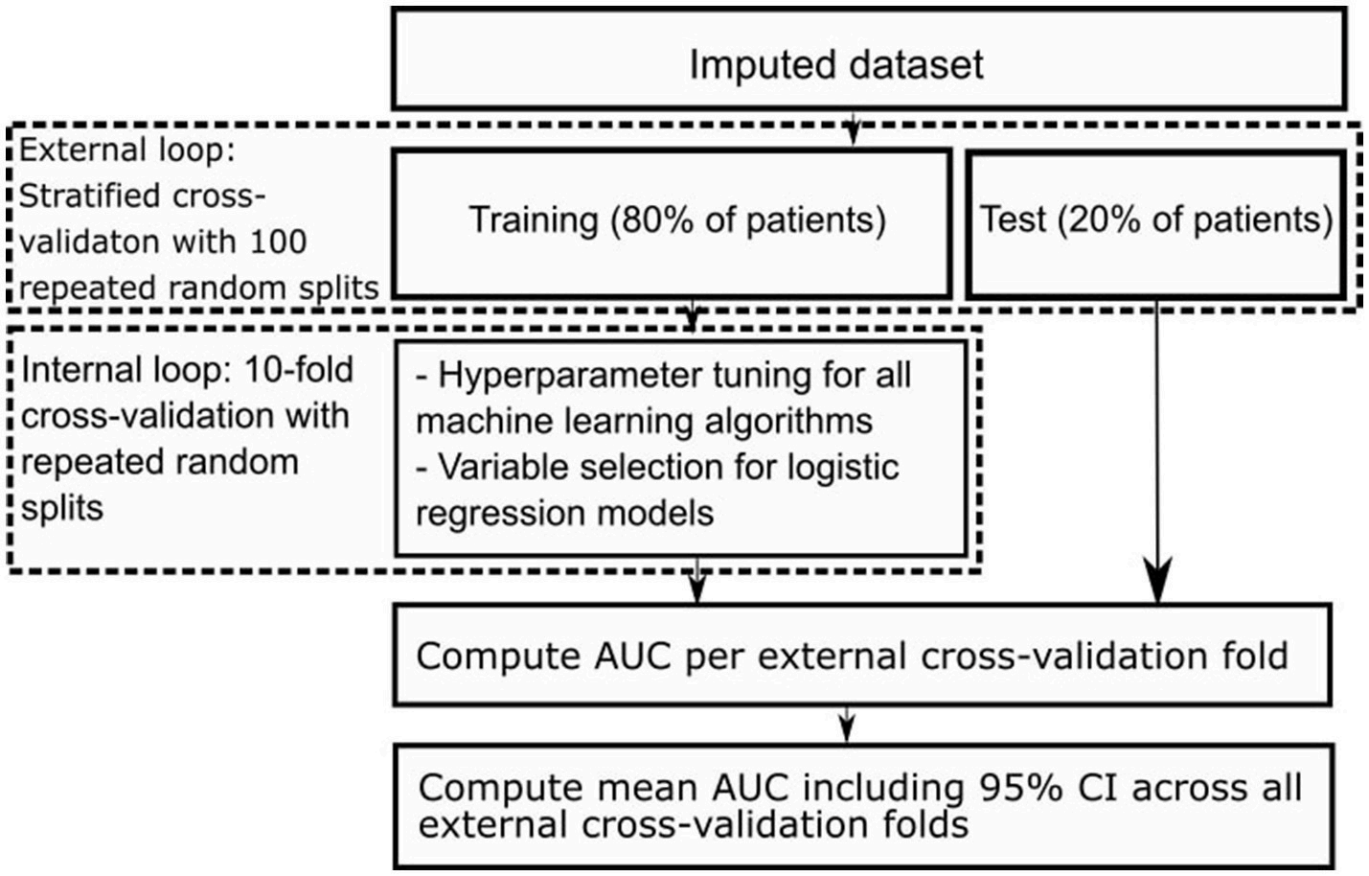
Schematic representation of nested cross-validation methodology.

We used nested cross-validation (CV), consisting of an outer and an inner CV loop. In the outer CV loop we used stratified CV with 100 repeated random splits resulting in a training set including 80% and a test set including 20% of all patients. Each training set was used as input for the inner CV loop, consisting of 10-fold CV (31, 32). In the inner CV loop we selected variables for the logistic regression models using the different variable selection methods, and optimized hyperparameters of all machine learning models. Hyperparameters are tuning parameters specific to each machine learning algorithm whose values have to be preset and cannot be directly learned from the data. We optimized hyperparameters with the random grid search module from Scikit-Learn (18). We selected those with highest area under the receiver operating characteristic (AUC) across all internal CV folds to find the best set of selected variables and hyperparameters. Figure 1 shows a schematic representation of our nested CV methodology.

For all Random Forests models of both prediction settings we ranked variables by decreasing variable importance. For each variable we assessed the frequency of being among the 15 most important variables in a Random Forests model for each of the 100 external CV folds (**Table 3**).

### Model performance

We assessed model discrimination (the ability to differentiate between patients with good and poor outcome) with receiver operating characteristic (ROC) analyses. Because of our outer CV loop with 100 repeated random splits, we obtained 100 different AUCs from every model. We computed the average ROC-curve and mean AUC with 95% confidence intervals (CI) for all models. We evaluated differences between mean AUCs of the best performing machine learning model and best performing logistic regression model by computing the difference of means including the associated 95% CI.

## Results

Of the 1,627 patients registered between March 2014 and June 2016, we excluded 244 patients for this analysis because of age <18 (*n* = 2), posterior circulation stroke (*n* = 79), missing MR CLEAN trial center (*n* = 20), and missing mRS or post-mTICI (*n* = 143). Mean age was 69.8 years (SD ± 14.4) and 738 (54%) of the 1,383 included patients were men. In total, 531 (38%) patients had good reperfusion after EVT and 525 (38%) were functionally independent (mRS ≤ 2) 3 months after stroke. Baseline characteristics are shown in Table 1.

**Table 1:** Baseline characteristics of participants.

**Characteristics**	**All patients (*n* = 1,383)**
Mean age ± SD *(years)*	69.8 ± 14.4
Men, *n (%)*	738 (53.5)
NIHSS score, median (IQR)^*^	16 (11–20)
Mean systolic blood pressure ± SD *(mm Hg)*	150 ± 25
**MEDICAL HISTORY**, ***N (%)***	
Atrial fibrillation	411 (30.7)
Hypertension	697 (51.1)
Diabetes mellitus	235 (17.1)
Myocardial infarction	216 (15.9)
Peripheral artery disease	127 (9.4)
Ischaemic stroke	227 (16.5)
Hypercholesterolemia	411 (29.7)
Pre-stroke mRS > 2, *n (%)*	158 (11.6)
Smoking, *n (%)*	314 (22.9)
**MEDICATION USE**, ***N (%)***	
DOAC^**^	35 (2.6)
Coumarine	179 (13.0)
Antiplatelet	461 (33.7)
Heparin	52 (3.8)
Blood pressure medication	707 (52.1)
Statin	490 (36.2)
Intravenous alteplase treatment, *n (%)*	1,054 (76.2)
ASPECTS, *median (IQR)*	9 (7–10)
Time from stroke onset to groin in minutes, *median (IQR)*	210 (160–270)
Collateral score ≥ 2	764 (55)

* National Institutes of Health Stroke Scale score.

** *Direct oral anticoagulant drugs*.

### Prediction of good reperfusion after EVT in patients at time of admission

Discrimination between good and poor reperfusion of the best machine learning algorithm (Support Vector Machine, mean AUC: 0.55) and the best logistic regression model (using backward elimination, mean AUC: 0.57) was similar (difference of mean AUCs: 0.02; 95% CI: 0.01–0.03). Discrimination was poor for all models, with a mean AUCs ranging from 0.53 to 0.57 (Table 2). Variable selection using LASSO or Elastic Net was not possible likely because the signal-to-noise ratio was insufficient (18).

**Table 2:** Discrimination of machine learning algorithms and logistic regression models across the various prediction settings.

Models, AUC (95% CI)^*^	**Prediction setting (used variables: predicted outcome)**		
	**Baseline: post-mTICI**	**Baseline: mRS**	**All variables: mRS**
Super learner	0.55 (0.54–0.56)	0.79 (0.79–0.80)	0.90 (0.90–0.91)
Random forests	0.55 (0.55–0.56)	0.79 (0.79–0.79)	0.91 (0.90–0.91)
Support vector machine	0.53 (0.53–0.54)	0.78 (0.77–0.78)	0.88 (0.88–0.89)
Neural network	0.53 (0.53–0.54)	0.77 (0.76–0.77)	0.88 (0.88–0.89)
LR: AUTOMATED SELECTION^**^			
Random forests	0.55 (0.55–0.56)	0.78 (0.78–0.78)	0.90 (0.90–0.90)
LASSO	NA^¥^	0.78 (0.78–0.79)	0.90 (0.89–0.90)
Elastic net	NA^¥^	0.77 (0.77–0.78)	0.89 (0.88–0.89)
Backward elimination	0.57 (0.57–0.58)	0.78 (0.77–0.78)	0.90 (0.89–0.90)
LR: prior knowledge^‡^	0.55 (0.55–0.58)	0.78 (0.78–0.79)	0.90 (0.90–0.90)

* Model discrimination is assessed by calculating mean Area Under the Curve (AUC) of the receiver operating characteristic across all outer cross-validation folds.

** Logistic regression using automated variable selection methods.

¥ Variable selection not possible, likely due to insufficient signal-to-noise ratio.

‡ *Logistic regression using variables based on prior knowledge*.

### Prediction of 3-months functional independence in patients at time of admission

Discrimination of good functional outcome of the best machine learning algorithm (Super Learner, mean AUC: 0.79) and the best logistic regression model (using LASSO, mean AUC: 0.78) was similar (difference of mean AUCs: 0.01; 95% CI: 0.01–0.01).

Discrimination was moderate for all models, with a mean AUCs ranging from 0.77 to 0.79.

### Prediction of 3-months functional independence in patients after performance of EVT

Discrimination of good functional outcome of the best machine learning algorithm (Random Forests, mean AUC: 0.91) and the best logistic regression model (using prior knowledge, mean AUC: 0.90) was similar (difference of mean AUCs: 0.01; 95% CI: 0.00–0.01).

Discrimination was good for all models, with mean AUCs ranging from 0.88 to 0.91.

We performed a *post-hoc* analysis in patients with good reperfusion as defined by post-mTICI ≥ 2b, predicting 3-months functional outcome both at time of admission and after performance of EVT. We did not find significant differences in performance between machine learning algorithms and logistic regression models in this patient subset (data not shown).

In Table 3 we show the top 15 variables based on the frequency of being among the 15 most important variables in a Random Forests model for each of the 100 external CV folds.

**Table 3:** Variable importance of Random Forests for various prediction settings (used variables: predicted outcome).

**Baseline: post-mTICI**	Freq^*^	**Baseline: mRS**	**Freq**	**All variables: mRS**	**Freq**
RR systolic at admission	100	Age	100	NIHSS after 24–48 h	100
Duration stroke onset to groin	100	NIHSS at baseline	100	Delta NIHSS: follow-up minus baseline	100
RR diastolic at admission	100	Duration stroke onset to groin	100	Age	100
Thrombocyte count	100	Glasgow Coma Scale	100	NIHSS at baseline	100
Age	100	RR systolic at admission	100	Duration from onset to recanalization	100
Creatinine	100	CRP	100	Duration of procedure	100
CRP	100	Creatinine	100	Delta NIHSS ≥ 4 points higher after EVT	100
NIHSS at baseline	100	Thrombocyte count	100	Duration stroke onset to groin	100
Clot burden score	100	RR diastolic at admission	100	Glasgow Coma Scale	100
Glasgow ComaScale	100	mRS prior to stroke	100	Creatinine	100
ASPECTS score at baseline	100	ASPECTS score at baseline	100	CRP	100
Glucose	100	Glucose	100	Thrombocyte count	100
Location: proximal M1^**^	74	Clot burden score	99	RR systolic at admission	100
Hyperdense artery sign on NCCT	50	Presence of leukoaraiosis	96	mRS prior to stroke	91
History of atrial fibrillation	32	Collateral score	77	RR diastolic at admission	93

NCCT, non-contrast CT; CRP, C-Reactive Protein; RR, blood pressure; NIHSS, National Institutes of Health Stroke Scale score.

* *Frequency of being among the 15 most important variables in a Random Forests model for each of the 100 external CV folds*.

** *Location of intracranial occlusion on CTA*.

## Discussion

We found no difference in performance between best performing machine learning algorithms and best performing logistic regression models in predicting radiological or clinical outcome in stroke patients treated with EVT. For prediction of good reperfusion using variables available at baseline, all models showed a poor discriminative performance. This could indicate that reperfusion after EVT depends on characteristics not present in our variables available at time of admission, such as vascular anatomy or interventionalist related factors. Prediction of 3-months functional independence using variables known at baseline was moderate, predicting 3-months functional independence using baseline and treatment variables resulted in good performance.

We hypothesized that machine learning would outperform logistic regression models due to simultaneous assessment of a large number of variables, and more efficient processing of non-linear relations and interactions between them. Although a large number of variables (83 in total, see Supplementary Tables II, III) were available for analysis in the MR CLEAN Registry database, performance of best machine learning algorithms and best logistic regression models were similar. This could indicate that interactions and non-linear relationships in our dataset were of limited importance.

To interpret our results, several methodological limitations have to be considered. First, due to their great flexibility machine learning algorithms are prone to overfitting, which results in optimistic prediction performance. To account for overfitting we used nested CV, which is considered to be an effective method for this aim (33). Second, our outer CV loop resulted in 100 AUCs per model leading to relatively small confidence intervals of mean AUCs. Although this increases the probability of statistically significant differences between mean AUCs of various models, the clinical relevance of these mean AUC differences is difficult to interpret. Because in our study mean AUC differences between models are minimal, clinical relevance of these differences is also negligible. Third, we used data from a registry. Registries might be prone to selection bias. However, we expect that selection bias in our study was minimal because the MR CLEAN Registry in principle covers all patients treated with EVT in the Netherlands. In addition, in all centers patients were treated according to national guidelines, and registration of treatment was a prerequisite for reimbursement (11).

Strong points of this study include the large sample size and standardized collection of patient data. Moreover, because of extensive hyperparameter tuning and state-of-the art variable selection methods, machine learning and logistic regression models were compared at their best performance. In several other studies that compared machine learning algorithms with only logistic regression methods using variables based on prior knowledge, machine learning outperformed logistic regression (6, 7, 34). Variable selection based on prior knowledge has the major drawback that predictive patterns in the data may be missed, as variable selection is strictly based on the literature and expert opinion (26). In our study however, logistic regression using variables based on prior knowledge performed similarly to logistic regression using automated variable selection methods.

The distinction between machine learning and “classical” regression methods is largely artificial. However, a clear distinction between various machine learning algorithms and logistic regression exists in terms of model transparency, which could be seen as the understanding of the mechanism by which the model works (35). Logistic regression has the advantage of transparency at the level of individual variable coefficients, since from these coefficients odds ratios can be derived. However, variable importances derived from the Random Forests algorithm also offer insight in the importance of individual variables for prediction performance (22). These variable importances take interaction between variables into account and have a similar interpretation for continuous and discrete variables, unlike odds ratios which constitute an effect per unit change of a predictor. Hence, Random Forests could be used as an efficient screening tool to pick up predictive patterns in the data that could potentially lead to further hypothesis-driven research. In Table 3 we show the top 15 variables from either the baseline or baseline and treatment variable set, based on Random Forests variable importance. The majority of variables in Table 3 do not overlap with the selection of variables based on prior knowledge, potentially providing researcher with additional information.

In this dataset we found no clinically relevant differences in prediction of reperfusion and 3-months functional independence across all models. However, since it is generally not known on beforehand which type of model will result in the best predictive performance in a new dataset, our methodology could be of importance in future studies. We present an analysis pipeline with both machine learning algorithms and logistic regression models including state-of-the-art variable selection methods. Assessing predictive performance of all models simultaneously enables the researcher to make the proper trade-off between predictive performance and model transparency. As our analysis pipeline is fully automated and input variables and outcome label can be altered at will, it is relatively easy to reuse in future studies. The Python code of our pipeline has been made publicly available in an online repository (https://github.com/L-Ramos/MrClean_Machine_Learning).

## Ethics statement

The central medical ethics committee of the Erasmus Medical Centre Rotterdam, the Netherlands, evaluated the MR CLEAN Registry protocol and granted permission to carry out the study as a registry. All subjects gave written informed consent in accordance with the Declaration of Helsinki.

## Author contributions

HvO lead author, study design, analysis and interpretation, critical revision manuscript for important intellectual content. LR study design, analysis and interpretation, critical revision of manuscript for important intellectual content. AH, MvL, ES, HL, SO, KZ, EV, and HM study design, critical revision of manuscript for important intellectual content. NK data acquisition, critical revision of manuscript for important intellectual content. DD, MvW, IvdS, WS, and CM data acquisition, critical revision of manuscript for important intellectual content. MW supervisor of lead author, data acquisition, study design, critical revision of manuscript for important intellectual content.

### Conflict of interest statement

DD reports grants from the Dutch Heart Foundation, AngioCare, Medtronic/Covidien/EV3, MEDAC/LAMEPRO, Penumbra, Top Medical/Concentric, and Stryker during conduct of the study; grants from Stryker European Operations BV, Medtronic, Dutch Heart Foundation, Brain Foundation Netherlands, The Netherlands Organisation for Health Research and Development, Health Holland Top Sector Life Sciences and Health, and consultation fees from Stryker, Bracco Imaging, and Servier, received by the Erasmus University Medical Centre, outside the submitted work. CM reports grants from TWIN, during the conduct of the study and grants from CVON/Dutch Heart Foundation, European Commission and from Stryker outside the submitted work (paid to institution), and is shareholder of Nico.lab. HM is founder and shareholder of Nico-lab. The remaining authors declare that the research was conducted in the absence of any commercial or financial relationships that could be construed as a potential conflict of interest.
