# Supplementary material for: Predicting Outcome of Endovascular Treatment for Acute Ischemic Stroke: Potential Value of Machine Learning Algorithms
Source: Front Neurol. 2018 Sep 25;9:784. doi: 10.3389/fneur.2018.00784 (PMC6167479; doi:10.3389/fneur.2018.00784)
Supplement: Supplementary file 1 [file Table_1.docx]

| **Supplementary Table I. Variables selected for logistic regression based on prior knowledge** | |
| --- | --- |
|  |  |
| **Baseline variables (n = 13)** | **Baseline and treatment variables (n = 15)** |
| Age | Age |
| mRS prior to stroke | mRS prior to stroke |
| History of diabetes mellitus | History of diabetes mellitus |
| History of previous ischemic stroke | History of hypertension |
| History of atrial fibrillation | History of previous ischemic stroke |
| Systolic blood pressure | Systolic blood pressure |
| Intravenous thrombolysis | Intravenous thrombolysis |
| Collateral score on CTA | Collateral score on CTA |
| Location of intracranial occlusion on CTA | Time from onset stroke to groin |
| ASPECTS score on baseline | Duration of EVT procedure |
| NIHSS at baseline | Location of intracranial occlusion on DSA |
| Duration stroke onset stroke to groin | mTICI post EVT |
| Clot burden score on CTA | NIHSS post EVT (24-48 hours) |
|  | General anesthesia during EVT |
|  | Symptomatic intracerebral hemorrhage |
|  |  |
| mRS = modified Rankin Scale; CTA = CT angiography; DSA = Digital Substraction Angiography; mTICI = modified Thrombolysis in Cerebral Infarction score; AOL = Arterial Occlusive Lesion recanalization score; NIHSS = National Institutes of Health Stroke Scale score; | |
|  |  |
|  |  |
