# Supplementary material for: Predicting Outcome of Endovascular Treatment for Acute Ischemic Stroke: Potential Value of Machine Learning Algorithms
Source: Front Neurol. 2018 Sep 25;9:784. doi: 10.3389/fneur.2018.00784 (PMC6167479; doi:10.3389/fneur.2018.00784)
Supplement: Supplementary file 2 [file Table_2.DOCX]

| **Supplementary Table II. Variables available at baseline** | |
| --- | --- |
|  |  |
| **Variables (n = 53)** |  |
| Age | Non-contrast CT |
| Sex | Hyperdense artery sign |
| Medical history | Relevant (new) ischemia/ hypodensity |
| Stroke | Hemorrhagic transformation |
| Myocardial infarction | Leukoariosis |
| Peripheral artery disease | Old infarcts in same ASPECTS region |
| Diabetes mellitus | ASPECTS score |
| Hypertension | CT angiography |
| Atrial fibrillation | Intracranial atherosclerosis |
| Hypercholesterolemia | Vascular malformation/ aneurysm |
| mRS prior to stroke | Most proximal occlusion segment |
| Medication use | Collateral score |
| Antiplatelet use | Clot burden score |
| DOAC use | Symptomatic carotid bifurcation |
| Coumarine use | Stenosis |
| Heparin use | Atherosclerotic occlusion |
| Blood pressure medication | Floating thrombus |
| Statin use | Pseudo-occlusion |
| RR systolic | Carotid dissection |
| RR diastolic | NIHSS at baseline |
| Laboratory parameters | Admission on weekend |
| INR | Admission during off hours |
| Thrombocyte count | Transfer from other hospital |
| Creatinine | Intravenous thrombolysis |
| CRP | Glasgow Coma Scale |
| Glucose | Duration from onset to groin in minutes |
| Smoking |  |
|  |  |
| NIHSS = National Institutes of Health Stroke Scale score; CRP: C-Reactive Protein; INR: International Normalized Ratio; mRS = modified Rankin Scale; DOAC = Direct Oral Anticoagulant drugs | |
|  |  |
|  |  |
