# Supplementary material for: Predicting Outcome of Endovascular Treatment for Acute Ischemic Stroke: Potential Value of Machine Learning Algorithms
Source: Front Neurol. 2018 Sep 25;9:784. doi: 10.3389/fneur.2018.00784 (PMC6167479; doi:10.3389/fneur.2018.00784)
Supplement: Supplementary file 3 [file Table_3.DOCX]

| **Supplementary Table III. Variables availabe during and after EVT** | |
| --- | --- |
|  |  |
| **Variables (n = 30)** |  |
| Most proximal occlusion segment on DSA | Stent placement in ICA |
| Occlusion other territories | Balloon angioplasty |
| Used balloon guiding | Evidence of vascular injury on DSA |
| Complication during intervention | Duration from onset to recanalization |
| Performed procedure | Duration of procedure |
| First used EV treatment | General anesthesia |
| Attempts with MERCI as first choice | Conscious sedation |
| Administration of EVT medication | Reperfusion during EVT |
| Hemicraniectomy | IC stay |
| mTICI score pre EVT | High care stay |
| mTICI score post EVT | Stroke care stay |
| Occlusion side on DSA | Delta NIHSS: follow-up minus baseline |
| Pre-EVT AOL score | Delta NIHSS ≥ 4 points higher after EVT |
| Post-EVT AOL score | Symptomatic intracranial hemorrhage |
| Total attempts | NIHSS after 24-48 hours |
|  |  |
| EVT = Endovascular Treatment; DSA = Digital Substraction Angiography; mTICI = modified Thrombolysis in Cerebral Infarction score; AOL = Arterial Occlusive Lesion recanalization score; NIHSS = National Institutes of Health Stroke Scale score | |
|  |  |
|  |  |
