# Supplementary material for: Predicting Outcome of Endovascular Treatment for Acute Ischemic Stroke: Potential Value of Machine Learning Algorithms
Source: Front Neurol. 2018 Sep 25;9:784. doi: 10.3389/fneur.2018.00784 (PMC6167479; doi:10.3389/fneur.2018.00784)
Supplement: Supplementary file 4 [file Table_4.docx]

ACKNOWLEDGEMENTS

Rotterdam, March 27, 2018

Executive committee

Diederik W.J. Dippel^1^; Aad van der Lugt^2^; Charles B.L.M. Majoie^3^; Yvo B.W.E.M. Roos^4^; Robert J. van Oostenbrugge^5^; Wim H. van Zwam^6^; Jelis Boiten^14^; Jan Albert Vos^8^

Study coordinators

Ivo G.H. Jansen^3^; Maxim J.H.L. Mulder^1,2^; Robert- Jan B. Goldhoorn^5,6^

Local principal investigators

Wouter J. Schonewille^7^; Jan Albert Vos^8^; Charles B.L.M. Majoie^3^; Jonathan M. Coutinho^4^; Marieke J.H. Wermer^9^; Marianne A.A. van Walderveen^10^; Julie Staals^5^; Wim H. van Zwam^6^; Jeannette Hofmeijer^11^; Jasper M. Martens^12^; Geert J. Lycklama à Nijeholt^13^;Jelis Boiten^14^; Bob Roozenbeek^1^;Bart J. Emmer^2^; Sebastiaan F. de Bruijn^15^; Lukas C. van Dijk^16^; H. Bart van der Worp^17^; Rob H. Lo^18^; Ewoud J. van Dijk^19^; Hieronymus D. Boogaarts^20^; Paul L.M. de Kort^21^; Jo J.P. Peluso^26^; Jan S.P. van den Berg^22^; Boudewijn A.A.M. van Hasselt^23^; Leo A.M. Aerden^24^; René J. Dallinga^25^; Maarten Uyttenboogaart^28^; Omid Eshghi^29^; Tobien H.C.M.L. Schreuder^30^; Roel J.J. Heijboer^31^; Koos Keizer^32^; Lonneke S.F. Yo^33^; Heleen M. den Hertog^34^; Emiel J.C. Sturm^35^

Imaging assessment committee

Charles B.L.M. Majoie^3^(chair); Wim H. van Zwam^6^; Aad van der Lugt^2^; Geert J. Lycklama à Nijeholt^13^; Marianne A.A. van Walderveen^10^; Marieke E.S. Sprengers^3^; Sjoerd F.M. Jenniskens^27^; René van den Berg^3^; Albert J. Yoo^37^; Ludo F.M. Beenen^3^; Alida A. Postma^6^; Stefan D. Roosendaal^3^; Bas F.W. van der Kallen^13^; Ido R. van den Wijngaard^13^; Adriaan C.G.M. van Es^2^; Bart J. Emmer^2,3^; Jasper M. Martens^12^; Lonneke S.F. Yo^33^; Jan Albert Vos^8^; Joost Bot^36^, Pieter-Jan van Doormaal^2^.

Writing committee

Diederik W.J. Dippel^1^(chair); Aad van der Lugt^2^; Charles B.L.M. Majoie^3^; Yvo B.W.E.M. Roos^4^; Robert J. van Oostenbrugge^5^; Wim H. van Zwam^6^; Geert J. Lycklama à Nijeholt^13^; Jelis Boiten^14^; Jan Albert Vos^8^; Wouter J. Schonewille^7^; Jeannette Hofmeijer^11^; Jasper M. Martens^12^; H. Bart van der Worp^17^; Rob H. Lo^18^

Adverse event committee

Robert J. van Oostenbrugge^5^(chair); Jeannette Hofmeijer^11^; H. Zwenneke Flach^23^

Trial methodologist

Hester F. Lingsma^38^

Research nurses / local trial coordinators

Naziha el Ghannouti^1^; Martin Sterrenberg^1^; Corina Puppels^7^; Wilma Pellikaan^7^; Rita Sprengers^4^; Marjan Elfrink^11^; Joke de Meris^14^; Tamara Vermeulen^14^; Annet Geerlings^19^; Gina van Vemde^22^; Tiny Simons^30^; Cathelijn van Rijswijk^21^; Gert Messchendorp^28^; Hester Bongenaar^32^; Karin Bodde^24^; Sandra Kleijn^34^; Jasmijn Lodico^34^; Hanneke Droste^34^; M. Wollaert^5^; D. Jeurrissen^5^; Ernas Bos^9^; Yvonne Drabbe^15^; Marjan Elfrink^11^; Berber Zweedijk^17^; Mostafa Khalilzada^15^.

PhD / Medical students:

Esmee Venema^38^; Vicky Chalos^1,38^; Kars C.J. Compagne^2^; Ralph R. Geuskens^3^; Tim van Straaten^19^; Saliha Ergezen^1^; Roger R.M. Harmsma^1^; Daan Muijres^1^; Anouk de Jong^1^; Wouter Hinseveld^7^; Olvert A. Berkhemer^1,3,6^; Anna M.M. Boers^3,39^; J. Huguet^3^; P.F.C. Groot^3^; Marieke A. Mens^3^; Katinka R. van Kranendonk^3^; Kilian M. Treurniet^3^; Manon Kappelhof^3^; Manon L. Tolhuijsen^3^; Heitor Alves^3^.

List of affiliations

Department of Neurology^1^, Radiology^2^, Public Health^38^, Erasmus MC University Medical Center;

Department of Radiology^3^, Neurology^4^, Biomedical Engineering & Physics^39^, Academic Medical Center, Amsterdam;

Department of Neurology^5^, Radiology^6^, Maastricht University Medical Center and Cardiovascular Research Institute Maastricht (CARIM);

Department of Neurology^7^, Radiology^8^, Sint Antonius Hospital, Nieuwegein;

Department of Neurology^9^, Radiology^10^, Leiden University Medical Center;

Department of Neurology^11^, Radiology^12^, Rijnstate Hospital, Arnhem;

Department of Radiology^13^, Neurology^14^, MC Haaglanden, the Hague;

Department of Neurology^15^, Radiology^16^, HAGA Hospital, the Hague;

Department of Neurology^17^, Radiology^18^, University Medical Center Utrecht;

Department of Neurology^19^, Neurosurgery^20^, Radiology^27^, Radboud University Medical Center, Nijmegen;

Department of Neurology^21^, Radiology^26^, Sint Elisabeth Hospital, Tilburg;

Department of Neurology^22^, Radiology^23^, Isala Klinieken, Zwolle;

Department of Neurology^24^, Radiology^25^, Reinier de Graaf Gasthuis, Delft;

Department of Neurology^28^, Radiology^29^, University Medical Center Groningen;

Department of Neurology^30^, Radiology^31^, Atrium Medical Center, Heerlen;

Department of Neurology^32^, Radiology^33^, Catharina Hospital, Eindhoven;

Department of Neurology^34^, Radiology^35^, Medical Spectrum Twente, Enschede;

Department of Radiology^36^, VUMC, Amsterdam;

Department of Radiology^37^, Texas Stroke Institute, Texas, United States of America;
